# Development and validation of a novel scoring system for acute ischemic stroke

**DOI:** 10.1186/s12967-024-04967-5

**Published:** 2024-02-14

**Authors:** Rui Xu, Shixin Peng, Yulong Qiu, Ji Zhu, Xiaodong Zhang, Li Jiang

**Affiliations:** Department of Neurosurgery, The First Affiliated Hospital of Chongqing Medical University, No. 1, Youyi Road, Yuanjiagang, Yuzhong District, Chongqing, 400016 China

## Abstract

**Background:**

This study aimed to assess the clinical application of the Artery Occlusion Image Score (AOIS), a new metric based on computed tomographic angiography (CTA) that reflects the severity of occlusive changes in the main intracranial arteries.

**Materials and Methods:**

Patients diagnosed with acute ischemic stroke (AIS) were divided into three groups: anterior circulation infarcts (ACI group), posterior circulation infarcts (PCI group), and both anterior and posterior circulation infarcts (ACI + PCI group). The sensitivity and specificity of AOIS were evaluated using the Basilar Artery on Computed Tomography Angiography (BATMAN) score, the Clot Burden Score (CBS), and the National Institutes of Health Stroke Scale (NIHSS) as comparators through receiver-operating characteristic (ROC) curve analysis.

**Results:**

The final analysis included 439 consecutive patients. In the ACI group, AOIS demonstrated high sensitivity (86.3%) and specificity (85.0%) and outperformed CBS in predicting patient prognosis. In the PCI group, AOIS also showed high sensitivity (88.9%) and specificity (90.0%) and outperformed BATMAN in predicting patient prognosis. In the ACI + PCI group, AOIS positively correlated with the NIHSS score (Spearman’s ρ = 0.602, P < .001). Additionally, the scoring time of AOIS did not significantly differ from CBS and BATMAN.

**Conclusion:**

AOIS is a convenient and reliable method for guiding treatment and predicting outcomes in patients with ACI or/and PCI. Furthermore, AOIS is the first CTA-based scoring system that covers both the anterior and posterior circulation, providing a convenient and reliable evaluation for patients with concurrent acute ischemic stroke in both circulations.

## Introduction

Acute ischemic stroke (AIS) is a highly prevalent form of cerebrovascular disease that has serious adverse effects on quality of life, is associated with disability and mortality [1–3]. In China, the number of patients with AIS is likely to increase steadily because of the large aging population, the increasing prevalence of conventional risk factors, and inadequate management [4–6]. The economic burden of AIS on patients and their families is substantial, and it places a substantial strain on social resources.

Usually, the severity of AIS is closely related with the thrombus burden (the number, site, and extent of blood clots) [7–9]. Therefore, timely and accurate assessment of involved artery and appropriate interventions are crucial for improving the prognosis of AIS patient [10]. Computed tomography angiography (CTA) can provide rapid and high-resolution images of intracranial arteries and is now widely used to evaluate the degree of cerebral vascular patency in cerebrovascular accidents [11–13]. Thus, several scoring systems for predicting the prognosis of AIS patients [14, 15], such as Clot Burden Score (CBS) [16], Basilar Artery on Computed Tomography Angiography (BATMAN) [17], posterior circulation collateral score (PC-CS) [18, 19], posterior circulation CTA (PC-CTA) [20], were based on the images provided by the CTA, providing important clinical decision-making supports for doctors. However, due to the specific focus of these methods, they may not be universally applicable to all cases of AIS. For instance, the CBS is used to evaluate anterior circulation ischemic stroke, while the BATMAN, PC-CS and PC-CTA are used to assess posterior circulation ischemic stroke, both of which had limited applicability for assessing AIS involving simultaneously both anterior and posterior circulation. Furthermore, these methods usually focus on larger vessels of Wilis circle and had limited applicability for assessing vessels with partial filling defects.

Previously, we developed the Artery and Venous Sinus Occlusion Image Score (AVOIS) for occlusive cerebral artery and vein diseases, which is primarily designed to guide patient treatment by the degree of anterior circulation occlusion and forecasting patient prognosis [21]. The aim of this study was to develop a novel artery scoring method, the Artery Occlusion Image Score (AOIS), which the main difference between AOIS and AVOIS is that AOIS has the capability to simultaneously assess both anterior and posterior circulation, and verifying its clinical applicability for the prediction of AIS prognosis.

## Material and methods

### Patients

This retrospective study was approved by the ethical committees of the Hospital. We retrospectively screened 504 patients with AIS who were admitted to the Department of Neurology at the (blinded for review) Hospital in Chongqing, China from April 2019 to October 2020. 132 of the 504 patients have been previously reported. This prior article established an artery and venous sinus occlusion image score (AVOIS) which is compatible in both cerebral arteries and venous system diseases whereas in this manuscript we development and validation a novel scoring system to assess multiple infarcts involving both anterior and posterior circulation [21]. Inclusion criteria included: (i) age between 18 and 80 years, (ii) presentation of acute disabling neurological deficits (including motor disorders, sensory impairments, language disorders, cognitive impairments, etc.), (iii) a definite diagnosis of AIS, and (iv) availability of CTA data. Patients were excluded if they: (i) experienced AIS symptom onset more than 2 weeks prior to admission [13, 22]; (ii) had been diagnosed with a transient ischemic attack (TIA); or (iii) had other diseases that could affect the assessment of AIS, such as mental disorders, physical disabilities, epilepsy, and peripheral nerve diseases.

**Fig. 1:**
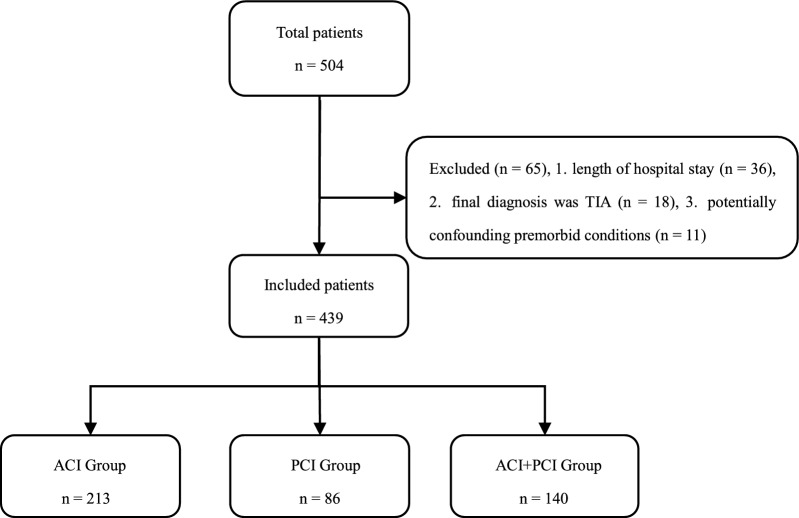
Flowchart of grouping. Flowchart shows participant enrollment in the study. TAI: transient ischemic attack

We divided patients with AIS into three groups according to the infarction site based on Damasio’s template mapping, the Bogousslavsky classification standard and the Oxfordshire Community Stroke Project (OCSP): an anterior circulation infarct (ACI) group, a posterior circulation infarct (PCI) group, and an ACI + PCI group [23–25] (Fig. 1).

### Imaging protocol and analysis

CTA was performed using a 64-multidetector row spiral CT machine (Somatom Sensation 64; Siemens Medical Systems), and image data were transferred to a dedicated workstation for storage and post-processing.

**Fig. 2:**
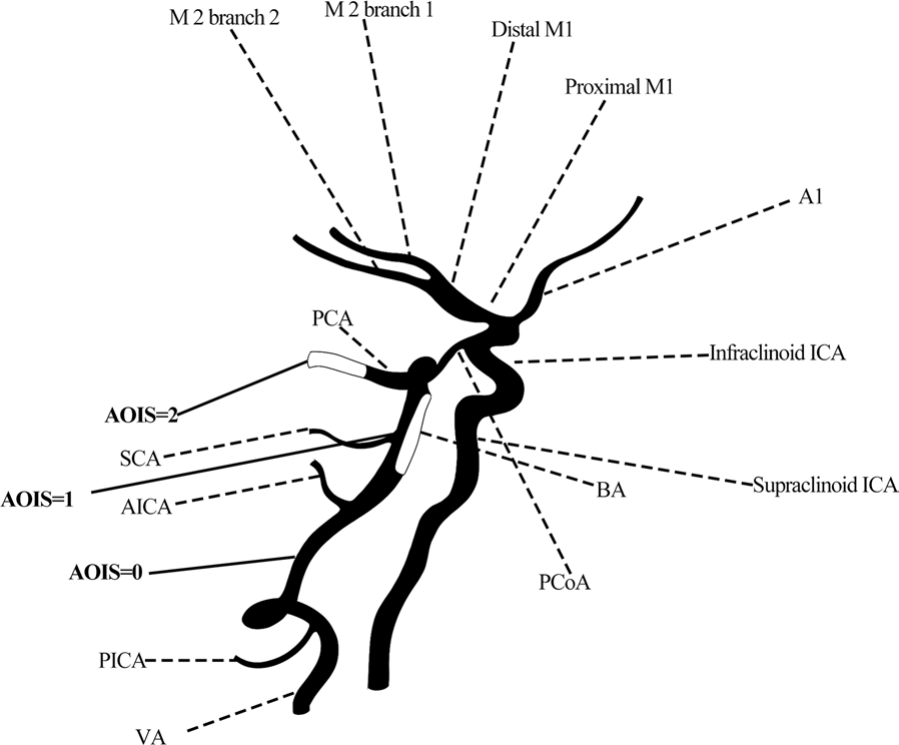
Artery Occlusion Image Score (AOIS). AOIS for acute ischemic stroke (AIS). The anterior and posterior circulations were assigned different scores (present = 0, partial occlusion = 1, absent = 2) by quantifying the thrombus

We developed AOIS as a semiquantitative CTA-based grading system to index the clot burden in the anterior and posterior circulation. AOIS is the first CTA score to simultaneously quantify the degree of anterior and posterior circulation occlusion in patients with ACI and PCI. In the ACI group, we referred to CBS to divide the anterior cerebral artery, middle cerebral artery, and internal carotid artery of the anterior circulation into seven segments and quantified their thrombus burden [16]. In the PCI group, based on BATMAN [17], we quantified the thrombus burden of the large vertebrobasilar vessel and the main vertebrobasilar side branches: the posterior inferior cerebellar artery (PICA), anterior inferior cerebellar artery (AICA), and superior cerebellar artery (SCA). Based on the CTA findings, two radiologists with more than 20 years working experience assessed the anterior and posterior circulation using a double-blind approach. They remained completely unaware of the patient's name, age, admission status, and prognosis, ensuring unbiased evaluation. In the event of a disagreement between the two researchers, the final verdict was made by a chief radiologist with more than 25 years working experience. Inter-rater reliability was assessed with kappa statistics. Scores were assigned according to the severity of intracranial artery occlusion, in which 0 = present, 1 = partial occlusion, and 2 = absent (Fig. 2, Table 1):

**Table 1:** Summary of the artery occlusion image score (AOIS)

	Present	Partial occlusion	Absent
Anterior circulation			
Infraclinoid ICA	0	1	2
Supraclinoid ICA	0	1	2
Proximal M1 segment	0	1	2
Distal M1 segment	0	1	2
M2 branch 1	0	1	2
M2 branch 2	0	1	2
A1 segment	0	1	2
Posterior circulation			
VA	0	1	2
BA	0	1	2
PICA	0	1	2
AICA	0	1	2
SCA	0	1	2
PCA	0	1	2
Pcom	0	1	2

*ICA* internal carotid artery; *M1* M1 segment of the middle cerebral artery; *M2* M2 segment of the middle cerebral artery; *VA* vertebral artery; *BA* basilar artery; *PICA* posterior inferior cerebellar artery; *AICA* anterior inferior cerebellar artery; *SCA* superior cerebellar artery; *PCA* posterior cerebral arteries; Pcom, posterior communicating artery

I. 2 points each were added for absence of contrast opacification in the complete cross-section of any part of the PCI and ACI.

II. 1 point was assigned to partial filling defects indicating stenosis or non-occlusive thrombus.

III. 0 point if either part intracranial artery was patent.

And classify AOIS into three levels based on the rating (Level I: < 5 points; Level II: 6–10 points; Level III: > 10 points).

In clinical practice, convenience and speed are critical factors that determine the practicality of scoring methods. To assess the clinical feasibility of the newly developed AOIS, we randomly selected 50 and 30 patients from the ACI and PCI groups, respectively, and measured the time required by the radiologists to complete the scoring process from CTA three-dimensional reconstruction to completion. This allowed us to compare the scoring time of the AOIS method with that of other established scoring methods.

### Outcome assessment

The modified Rankin Scale (mRS) score at 90 days was assessed by a stroke neurologist at our hospital's stroke center, who was blinded to the baseline NIHSS score, CT/CTA findings, and prognostic results [26]. The missing functional outcome data were imputed from the discharge mRS using the principle of carrying forward the last observed score. An mRS score > 2 was defined as a poor prognosis, and an mRS score ≤ 2 was defined as a good prognosis.

### Statistical analysis

Logistic regression was used to evaluate the association between the AOIS and poor prognosis (mRS > 2). We utilized tenfold cross-validation for internal validation, ensuring diverse training and evaluation subsets. External validation involved independent test datasets from other hospitals to assess the score's predictive ability on unseen data.

To explore the value of this novel score in the clinical prediction of patients with ACI, the AOIS was compared to the CBS. ROC curve analysis was then performed, with poor prognosis as the outcome variable and AOIS and CBS as the test variables. The area under the curve (AUC), sensitivity, and specificity of the two scores were compared. The Youden Index was utilized to determine the optimal cut-off value for the AOIS following the construction of a ROC curve. The DeLong test was used to determine whether the difference between the two groups was statistically significant.

In patients with PCI, the BATMAN score was used as a reference to evaluate the clinical value of the AOIS in predicting patient prognosis. ROC curve analysis was used to assess the prognostic performance, and the Youden Index was calculated to obtain the cut-off value for the AOIS after building a ROC. The AUCs were compared using the DeLong test.

In the ACI + PCI group, ROC curve analysis was used to determine the sensitivity and specificity of the AOIS for diagnosis. The National Institutes of Health Stroke Scale (NIHSS) is currently the most validated and widely used clinical rating instrument. In this study, we used the NIHSS score as a reference to evaluate the AOIS, using the Spearman correlation coefficient to assess the correlation between the NIHSS score and AOIS. *P* < 0.05 were considered statistically significant.

## Results

A total of 504 consecutive patients were identified. Of these, 65 were excluded—36 due to experiencing AIS symptom onset more than 2 weeks prior to admission; 18 with a final diagnosis of TIA, and 11 with potentially confounding premorbid conditions. The remaining 439 patients with AIS were included in the analysis, of whom 213 were diagnosed with ACI, 86 with PCI, and 140 with ACI + PCI.

### The ACI group

**Fig. 3:**
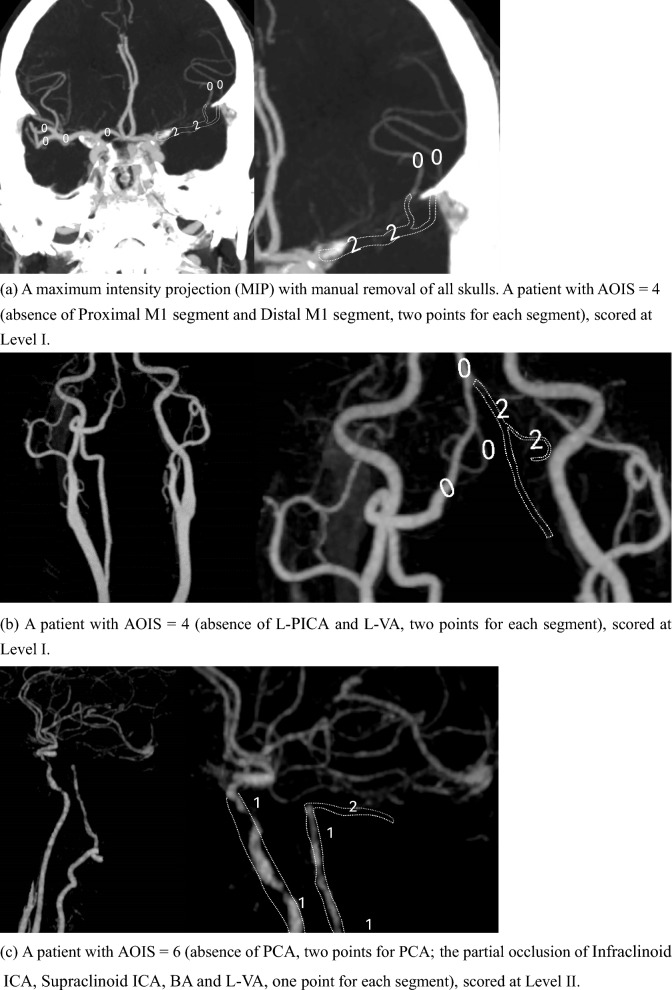
Scoring AIS patients by using AOIS

**Fig. 4:**
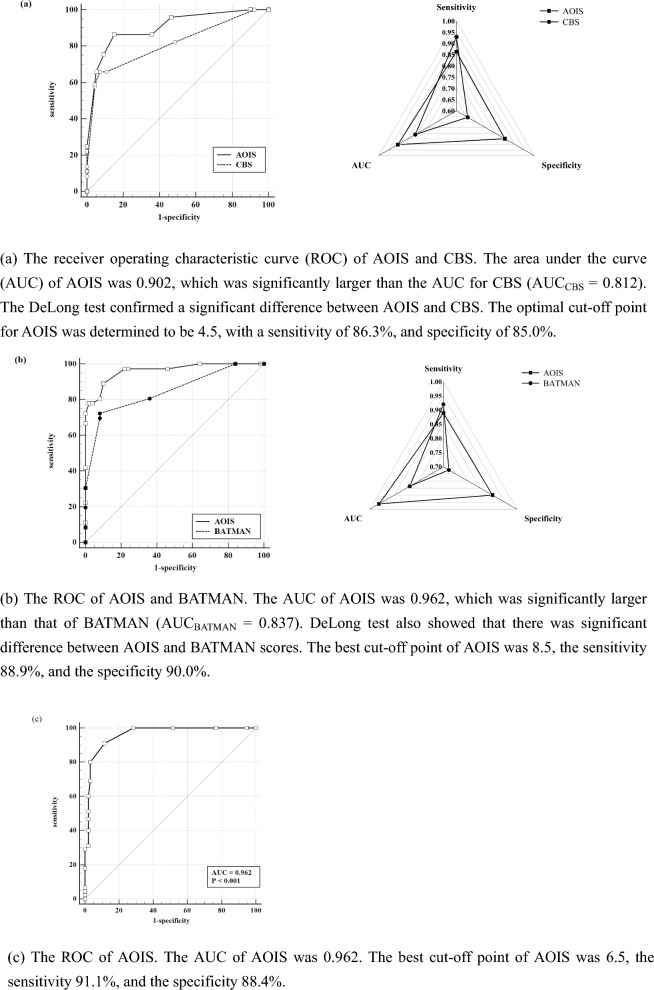
ROC curve analysis of AOIS, CBS and BATMAN Best Cutoff Value of clinical outcome

**Fig. 5:**
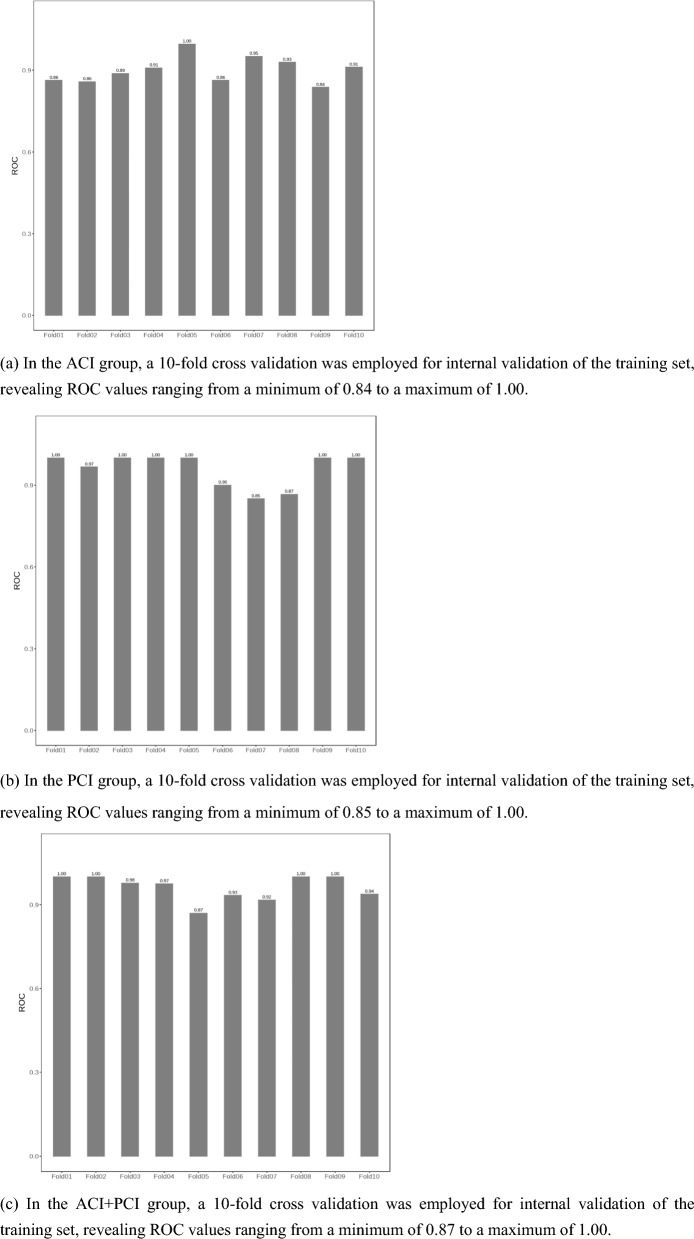
Training set tenfold cross validation results

**Fig. 6:**
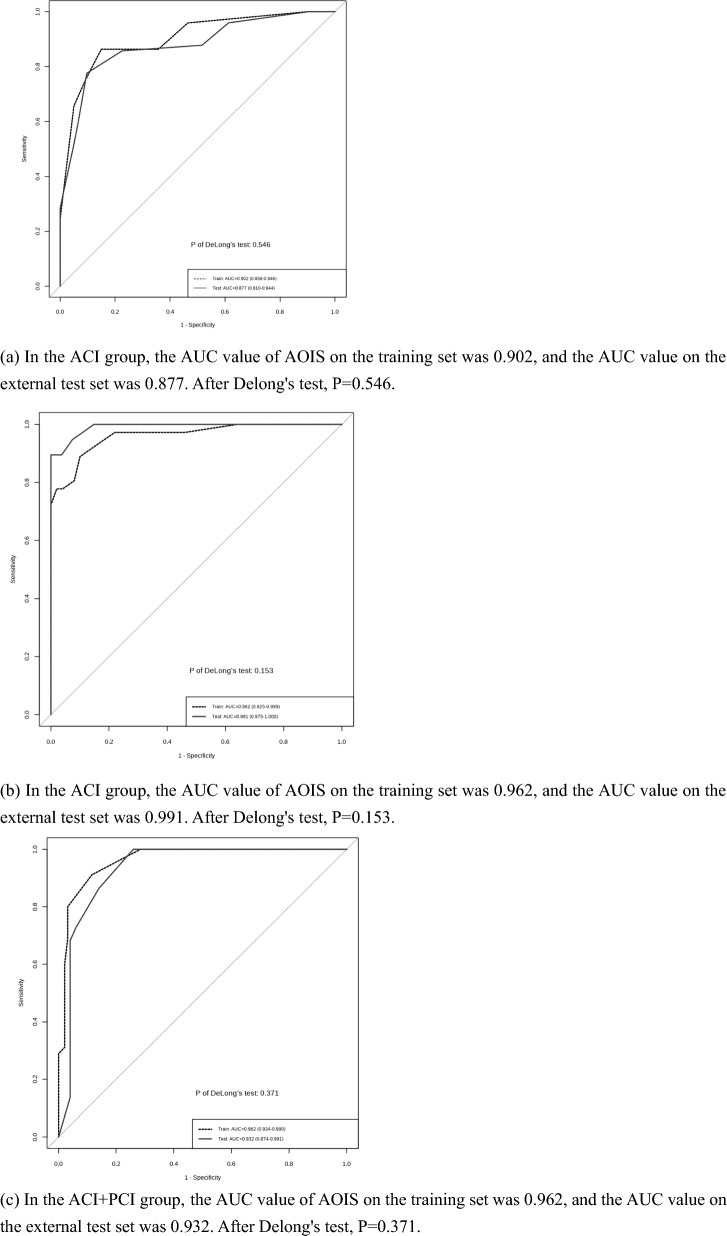
ROC curves of AOIS on training and external validation sets

The median age of the 213 patients with ACI, of whom 149 (70%) were men, was 64 (IQR: 57–71) years. Logistic regression showed significant differences in the prognosis according to atrial fibrillation (*P* = 0.002; OR 0.36; CI_95_ 0.19–0.68), and the CBS (*P* < 0.001; OR 0.49; CI_95_ 0.40–0.59), AOIS (*P* < 0.001; OR 2.11; CI_95_ 1.75–2.54), and NIHSS (*P* < 0.001; OR 1.49; CI_95_ 1.35–1.65) scores (Table 2). The AOIS (median [interquartile range (IQR)]; 2 [2–4] versus 8 [6–9]; *P* < 0.001) was lower in the good outcome group, and CBS (median [IQR]; 9 [8, 9] versus 4 [4–8]; *P* < 0.001) was higher in the good outcome group. This suggests that patients with higher AOIS scores or lower CBS scores are more likely to have good outcomes. In the ACI group, a significant positive correlation was found between AOIS and NIHSS scores (Spearman's ρ = 0.598, *P* < 0.001), indicating the heavier the thrombus burden in patients, the more severe their condition upon admission. To evaluate the value of AOIS in predicting the prognosis of patients with ACI, ROC curve analysis was used to test sensitivity and specificity. The AOIS showed high sensitivity (86.3%) and specificity (85.0%), and the best cutoff was 4.5. The AUC of the AOIS was 0.902, which was larger than that of the CBS (0.812). (Table 3) DeLong test showed that AOIS and CBS had statistical significance in predicting AUC and prognosis (z = 3.550, P < 0.001). Notably, AOIS yielded a Brier score of 0.059, outperforming CBS with a score of 0.061. These findings suggest that AOIS may exhibit superior prognostic capabilities in ACI patients (Figs. 3, 4a). AOIS achieved an AUC of 0.902 on the training set and 0.877 on the external test set. Delong test (P = 0.546) showed no significant AUC difference, indicating reliable external validation. Internal validation with tenfold cross-validation on the training set demonstrated consistently strong ROC values (0.84 to 1.00), confirming robust performance (Figs. 5, 6a).

**Table 2:** Comparison of baseline characteristics in three groups

ACI + PCI group	Over all, (*n* = 140)	Good Outcome, (*n* = 95)	Poor Outcome, (*n* = 45)	*P* Value
Age, median (IQR)	65 (56–72)	64 (56–71)	68 (61–72)	0.040
Male, n (%)	123 (88)	85 (89)	38 (85)	0.398
NIHSS, median (IQR)	4 (2–10)	3 (1–5)	15 (10–19)	< 0.001
AOIS, median (IQR)	6 (4–8)	5 (4–6)	11 (8–15)	< 0.001
Risk factors, n (%)				
Hypertension	98 (70)	66 (70)	32 (71)	0.843
Diabetes	53 (38)	39 (41)	14 (31)	0.259
Hyperlipidemia	47 (34)	34 (36)	13 (29)	0.420
Smoking	83 (60)	53 (56)	19 (67)	0.223
History of TIA or stroke	40 (29)	27 (28)	8 (29)	0.954
Atrial fibrillation	12 (9)	8 (8)	4 (9)	0.926
Coronary artery disease	22 (16)	14 (15)	5 (18)	0.645

PCI group	Over all, (n = 86)	Good Outcome, (n = 50)	Poor Outcome, (n = 36)	*P* Value
Age, median (IQR)	66 (55–72)	61 (53–70)	69 (64–72)	0.003
Male, n (%)	60 (70)	35 (70)	25 (70)	0.956
NIHSS, median (IQR)	5 (3–13)	3.5 (2–5)	13 (9–16)	< 0.001
BATMAN, median (IQR)	7 (4–8)	8 (7–8)	4 (3–7)	< 0.001
AOIS, median (IQR)	8 (5–14)	5 (4–6)	14 (12–16)	< 0.001
Risk factors, n (%)				
Hypertension	59 (69)	37 (74)	22 (61)	0.206
Diabetes	30 (35)	22 (44)	8 (22)	0.040
Hyperlipidemia	23 (27)	14 (28)	9 (25)	0.757
Smoking	36 (42)	25 (50)	11 (31)	0.074
History of TIA or stroke	17 (20)	11 (22)	6 (17)	0.541
Atrial fibrillation	22 (26)	11 (22)	11 (31)	0.371
Coronary artery disease	7 (8)	5 (10)	2 (5)	0.463

ACI group	Over all, (n = 213)	Good Outcome, (n = 140)	Poor Outcome, (n = 73)	*P* Value
Age, median (IQR)	64 (57–71)	63 (56–72)	67 (60–70)	0.264
Male, n (%)	149 (70)	96 (70)	53 (73)	0.543
NIHSS, median (IQR)	4 (2–12)	3 (2–4)	15 (9–18)	< 0.001
CBS, median (IQR)	8 (6–9)	9 (8–9)	4 (4–8)	< 0.001
AOIS, median (IQR)	4 (2–8)	2 (2–4)	8 (6–9)	< 0.001
Risk factors, n (%)				
Hypertension	155 (73)	102 (73)	53 (73)	0.968
Diabetes	59 (28)	44 (31)	15 (21)	0.094
Hyperlipidemia	39 (18)	26 (19)	13 (18)	0.700
Smoking	109 (51)	74 (53)	35 (48)	0.496
History of TIA or stroke	38 (18)	26 (19)	12 (16)	0.700
Atrial fibrillation	56 (26)	27 (19)	29 (40)	0.002
Coronary artery disease	42 (20)	30 (21)	12 (16)	0.386

statistically significant if *P* < 0 .05

*IQR* interquartile range; *NIHSS* National Institute of Health Stroke Scale; AOIS, Artery Occlusion Image Score; *TIA* transient ischemic attack

**Table 3:** ROC curve analysis of AOIS, CBS and BATMAN best cutoff value of clinical outcome

	ACI + PCI group	ACI group		PCI group	
	AOIS	AOIS	CBS	AOIS	BATMAN
AUC	0.962	0.902	0.812	0.962	0.837
Sensitivity	91.1%	86.3%	92.9%	88.9%	92.0%
Specificity	88.4%	85.0%	65.8%	90.0%	72.2%
Cutoff Value	6.5	4.5	6.5	8.5	6.5
DeLong Test	–	*Z* = 3.550, *P* < 0.001		*Z* = 3.547, *P* < 0.001	

statistically significant if *P* < 0.05

*AOIS* Artery Occlusion Image Score; *CBS* clot burden score; *BATMAN* The Basilar Artery on Computed Tomography Angiography Clot Burden Score

### The PCI group

The clinical characteristics of the 86 patients with PCI are summarized in Table 2. Overall, good outcomes were achieved in 58% (50/86) of the patients, and the median NIHSS score was 5 (IQR: 3–13). In the logistic regression analysis of PCI, older age (*P* = 0.003; OR 1.08; CI_95_ 1.03–1.15), diabetes (*P* = 0.040; OR 2.75; CI_95_ 1.05–7.21), NIHSS (*P* < 0.001; OR 1.39; CI_95_ 1.23–1.58), BATMAN (*P* < 0.001; OR 0.45; CI_95_ 0.33–0.61), and AOIS scores (*P* < 0.001; OR 2.06; CI_95_ 1.52–2.78) were associated with poor outcomes. However, sex and related risk factors (hypertension, hyperlipidemia, smoking, atrial fibrillation, TIA, coronary artery disease) were not associated with prognosis. A noteworthy positive correlation was observed between AOIS and NIHSS scores in the PCI group, with Spearman's correlation coefficient of 0.456 (*P* < 0.001), suggesting that with an increased thrombus burden, there was a corresponding escalation in the severity of the patient's condition upon admission. To test the accuracy of the AOIS in the prognosis of PCI, ROC curve analysis was used to test the sensitivity and specificity. The AOIS showed a specificity of 90.0%, sensitivity of 88.9%, and an optimal cutoff of 8.5. To further test the effectiveness and reliability of the AOIS in predicting the prognosis of patients with PCI, we used the BATMAN score as a reference. The AUC of the AOIS was 0.962, which was larger than that of the BATMAN (0.837). (Table 3) The DeLong test also revealed a significant difference between the AOIS and BATMAN scores. Meanwhile, AOIS demonstrated the Brier score of < 0.001, surpassing BATMAN, which scored 0.034, implying that the AIOS had an edge in predicting the prognosis of patients with PCI compared to BATMAN (Figs. 3, 4b). AOIS shows high consistency between the training set (AUC = 0.962) and external test set (AUC = 0.991), with no significant difference (p = 0.153). Internal validation via tenfold cross-validation on the training set confirms robust performance (ROC: 0.85 to 1.00) (Figs. 5, 6b).

### The ACI + PCI group

**Fig. 7:**
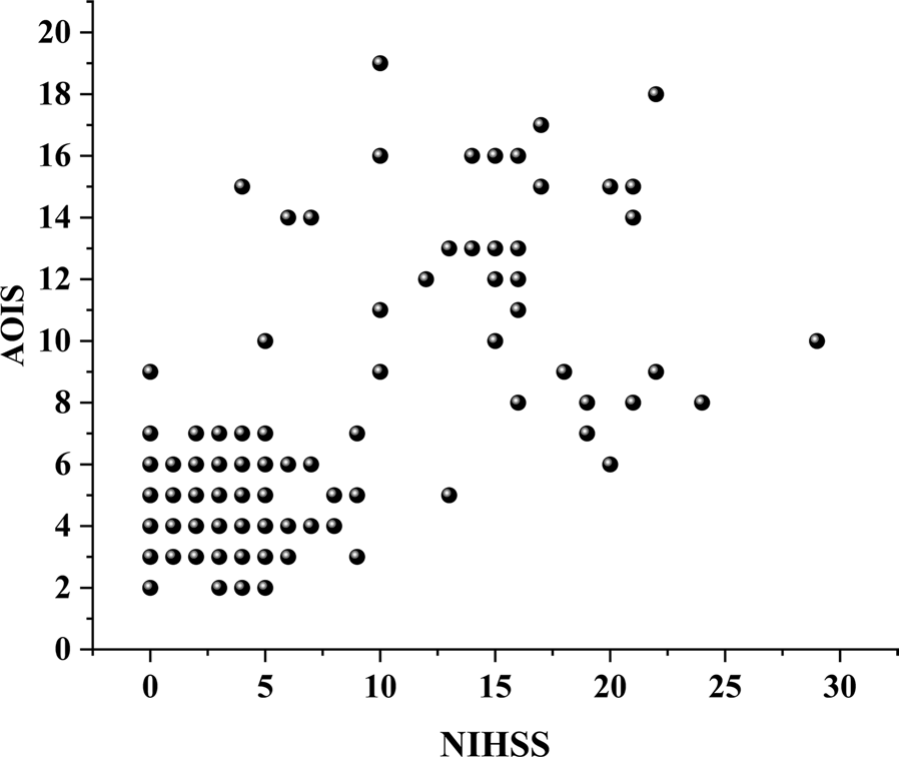
Positive correlation of AOIS and NIHSS. The National Institutes of Health Stroke Scale (NIHSS) score increased with the increase of AOIS score. AOIS was positively correlated with NIHSS (Spearman’s p = 0.602, p < .001). The higher the NIHSS score, the higher the AOIS score, and the greater the possibility of adverse outcomes

The median age of the 140 patients in the ACI + PCI group, of whom 123 (88%) were men, was 65 (IQR: 56–72) years. Overall, good outcomes were achieved in 67.9% (95/140) of the patients. The baseline characteristics are summarized in Table 2. The patients with good outcomes had lower median baseline NIHSS scores than those with poor outcomes (3 versus 15). The Spearman correlation test showed that the AOIS and NIHSS scores were associated (ρ = 0.602, *P* < 0.001), indicating the severity of patient's condition upon admission was associated with a heavier thrombus burden. Patients with a lower AOIS score (i.e., lower thrombus burden) had lower baseline NIHSS scores (Fig. 7). In the ACI + PCI group, AOIS showed high sensitivity (91.1%) and specificity (88.4%), (Table 3) and the best cutoff point was 6.5, suggesting that patients in the ACI + PCI group with AOIS > 6.5 were more likely to have a poor prognosis (Figs. 3, 4c). AOIS exhibits strong performance with AUC of 0.962 on training and 0.932 on external test. Delong test (*P* = 0.371) shows no significant AUC difference. Internal validation via tenfold cross-validation on training set indicates robust ROC values (0.87 to 1.00), confirming excellent performance.

### Time taken to calculate the score

**Fig. 8:**
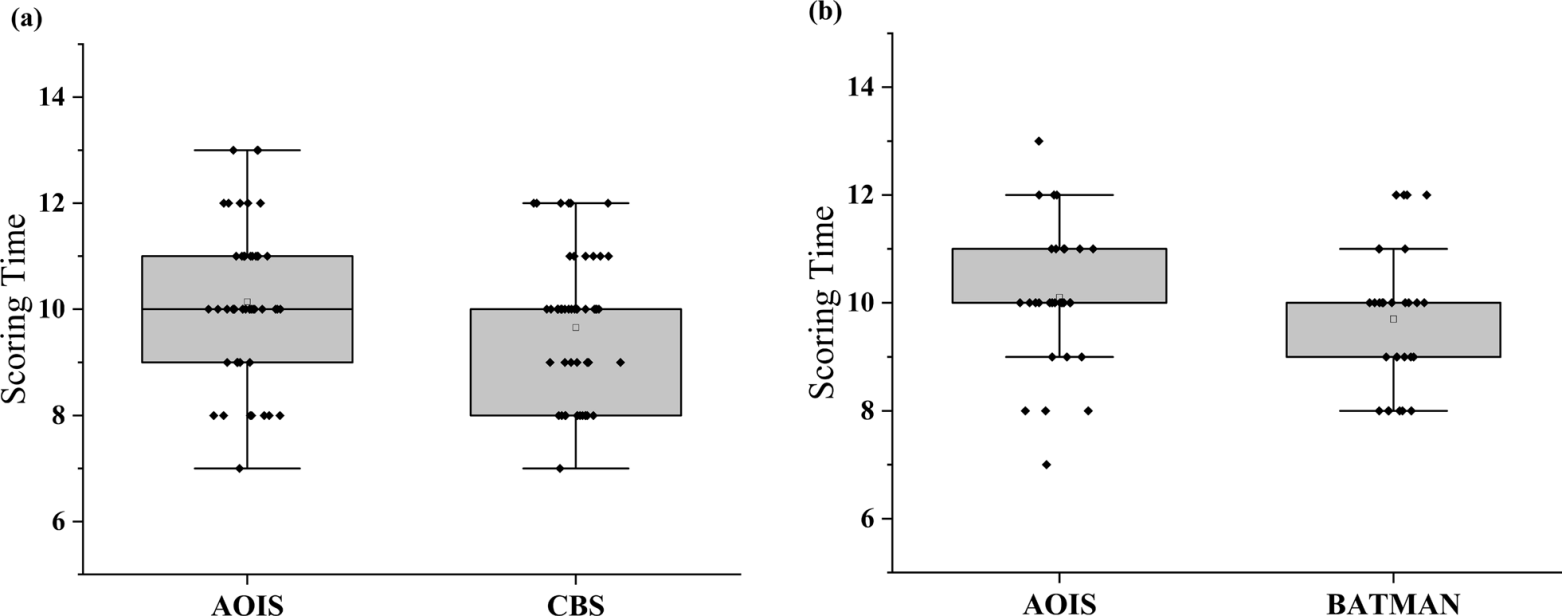
Comparison of scoring time. **a** In the ACI group, there was no significant difference in scoring time by using AOIS and CBS (p = .088). **b** In the PCI group, there was also no significant difference in scoring time by using AOIS and BATMAN (p = .0190)

In patients with ACI, the average length of time taken to score using the AOIS method was 10 min (standard deviation: 8.40–11.60 min), whereas the average time taken to score using the CBS method was 9 min (standard deviation: 7.60–10.41 min). For the PCI group, the mean scoring time using the AOIS was 10 min (standard deviation: 8.90–11.10 min), whereas the mean scoring time using BATMAN was 9.5 min (standard deviation: 8.35–10.65 min). The scoring time of the AOIS did not differ significantly from those of the CBS and BATMAN scores (Fig. 8).

## Discussion

In this study, we developed a novel CTA-based score, the AOIS, for the quantification of thrombus burden in patients with AIS. The results show that AOIS is an effective method of evaluating both anterior and posterior cerebral circulation. Furthermore, AOIS was accurate and effective in predicting outcomes in patients with multiple infarcts involving both ACI and PCI, provided a new scoring method for the assessment of the thrombus load in patients with multiple cerebral infarcts.

Various acute ischemic stroke scoring systems, such as NIHSS, ASPECTS, CBS, BATMAN, PC-CTA, and PC-CS, are utilized to assess patient prognosis based on clinical and imaging factors. While these tools play a crucial role in evaluating stroke severity, predicting outcomes, and guiding treatment decisions, they also exhibit limitations. Some systems emphasize neurological deficits, while others prioritize stroke size and imaging findings. Additionally, certain scoring methods may be overly complex, hindering swift assessments in emergency scenarios. Among numerous acute ischemic stroke scoring methods, CBS and BATMAN scores are based on CTA imaging and have been widely applied and validated in relevant research. Comparing these two scoring methods allows us to leverage existing research findings, enhancing the reference value of the comparison results.

Although many patients have multiple infarcts involving both anterior and posterior circulation, there is currently no system for vascular imaging scoring and predicting the prognosis of patients with simultaneous anterior and posterior circulation infarctions. To the best of our knowledge, the AOIS is the first system for scoring patients with both ACI and PCI based on CTA images. NIHSS is a well-known scale to evaluate the degree of neurological impairment in patients with AIS including ACI and/or PCI [27, 28], and its reliability and validity have been confirmed by clinical trials [29, 30]. Different from the NIHSS which is based on clinical symptoms not cerebral vascular images [31], the AOIS has some advantages in assessing the condition of AIS patient. First, the AOIS is not dependent on self-reporting of symptoms; therefore, it can also be used to score patients who are unconscious or unable to cooperate. Second, the AOIS is a CTA-based imaging scoring system, which is more objective and intuitive. Meanwhile, the results of the study showed a significant correlation between AOIS and NIHSS, suggesting NIHSS scores increased with the increasing AOIS scores. This meant that for patients in ACI + PCI group, an elevated AOIS score signified both a greater thrombus burden and more severe condition upon admission, and also suggested an unfavorable prognosis for the patient. The similar results were also found in the ACI and PCI group, indicating this innovative scoring tool could not only predict the prognosis of AIS patient follow-up, but also hold the potential to effectively grade the severity of PCI and ACI like NIHSS. Meanwhile, Internal and external validations affirm the AOIS strong predictive performance, validating its effectiveness., demonstrating AIOS was a reliable method for assessing the severity of multiple cerebral infarctions.

For the PCI group, the AOIS showed a high accuracy, sensitivity, and specificity in predicting the prognosis of PCI. Compared with anterior circulation infarction, patients with simple posterior circulation infarction are at greater risk of poor prognosis [32]. Previous studies have identified several clinical predictors of outcome following PCI, including the NIHSS score at admission, age, time to treatment, and recanalization [32, 33]. Meanwhile, the prognosis of the PCI patients may be also influenced by thrombus burden and collateral circulation compensation which can be assessed by the posterior circulation scoring methods such as PC-CTA, BATMAN, and PC-CS [17, 19]. However, these methods have some limitations, such as the need to evaluate large vessels and collateral branches separately, which could potentially lead to errors in partially occluded vessels. The AOIS in the study quantifies the thrombotic load in both the large vessels and the major collateral branches of the vertebrobasilar artery, providing more comprehensive coverage of the posterior circulation vessels and more elaborate scoring rules than the BATMAN score. After analyzing the AUC of the AOIS and BATMAN, we found the AOIS provided a more reliable and detailed assessment on the prognosis of PCI patients. At the same time, AOIS scoring method demonstrates consistent predictive efficacy through both internal and external validations.

The CBS are often used for assessing the patients’ anterior circulation [16]. Thus, the performance of AOIS was also compared with that of CBS. The results showed the AUC of AOIS was significantly higher than that of CBS, suggesting AOIS performed better than CBS in predicting the prognosis of ACI, particularly after accounting for partial filling defects of the anterior circulation.

In patients with AIS, scoring time is another important factor in determining whether scoring methods can be used for clinical purposes [34]. Notably, in comparison to CBS and BATMAN methods, AOIS didn't substantially extend score duration and didn't escalate the workload for clinical physicians. Moreover, AOIS provided more detailed information and improved prognostic accuracy compared to the CBS and BATMAN. This can aid in swift triage of AIS patients and serve as a guide for their treatment.

Although the results of this study are promising, it does have some limitations. First, the AOIS scoring system was highly dependent on image quality, and the scores of vessels that were not clearly displayed by CTA may be inaccurate. Second, this scoring system cannot be used to evaluate small branch vessels like anterior choroidal artery, pontine artery, which may impair its performance on predicting the prognosis of ASI patients. Third, this was a single-center study with a small sample size, which may cause some statistical biases and limit its generalizability.

In conclusion, the AOIS developed in this study is a convenient and reliable method for treatment guidance and outcome prediction in patients with ACI or/and PCI. Furthermore, the AIOS is the first CTA-based scoring system covering both anterior and posterior circulation, which provides convenient and reliable evaluations for patients with concurrent acute ischemic stroke in the anterior and posterior circulation.

## Data Availability

The data that support the findings of this study are not openly available due to reasons of sensitivity and are available from the corresponding author upon reasonable request.

## Abbreviations

ACI: Anterior circulation infarct
AOIS: Artery Occlusion Image Score
AVOIS: Artery and Venous Sinus Occlusion Image Score
BATMAN: Basilar Artery on Computed Tomography Angiography
CBS: Clot Burden Score
mRS: Modified Rankin Scale
NIHSS: National Institutes of Health Stroke Scale
PC-CS: Posterior circulation collateral score
PC-CTA: Posterior circulation computed tomography angiography
PCI: Posterior circulation infarct
